# Physical Determinants of Amyloid Assembly in Biofilm Formation

**DOI:** 10.1128/mBio.02279-18

**Published:** 2019-01-08

**Authors:** Maria Andreasen, Georg Meisl, Jonathan D. Taylor, Thomas C. T. Michaels, Aviad Levin, Daniel E. Otzen, Matthew R. Chapman, Christopher M. Dobson, Steve J. Matthews, Tuomas P. J. Knowles

**Affiliations:** a Department of Chemistry, University of Cambridge, Cambridge, United Kingdom; b Department of Life Sciences, Imperial College London, London, United Kingdom; c Paulson School of Engineering and Applied Sciences, Harvard University, Cambridge, Massachusetts, USA; d Interdisciplinary Nanoscience Center (iNANO), Aarhus University, Aarhus, Denmark; e Department of Molecular, Cellular, and Developmental Biology, University of Michigan College of Literature, Science, and the Arts, Ann Arbor, Michigan, USA; f Department of Physics, Cavendish Laboratory, Cambridge, United Kingdom; Duke University School of Medicine

**Keywords:** biofilms, functional bacterial amyloids, protein aggregation

## Abstract

A wide range of bacterial pathogens have been shown to form biofilms, which significantly increase their resistance to environmental stresses, such as antibiotics, and are thus of central importance in the context of bacterial diseases. One of the major structural components of these bacterial biofilms are amyloid fibrils, yet the mechanism of fibril assembly and its importance for biofilm formation are currently not fully understood. By studying fibril formation *in vitro*, in a model system of two common but unrelated biofilm-forming proteins, FapC from Pseudomonas fluorescens and CsgA from Escherichia coli, we found that the two proteins have a common aggregation mechanism. In both systems, fibril formation proceeds via nucleated growth of linear fibrils exhibiting similar measured rates of elongation, with negligible fibril self-replication. These similarities between two unrelated systems suggest that convergent evolution plays a key role in tuning the assembly kinetics of functional amyloid fibrils and indicates that only a narrow window of mechanisms and assembly rates allows for successful biofilm formation. Thus, the amyloid assembly reaction is likely to represent a means for controlling biofilm formation, both by the organism and by possible inhibitory drugs.

## Abstract (precis)

Biofilms are generated by bacteria, embedded in the formed extracellular matrix. The biofilm's function is to improve the survival of a bacterial colony through, for example, increased resistance to antibiotics or other environmental stresses. Proteins secreted by the bacteria act as a major structural component of this extracellular matrix, as they self-assemble into highly stable amyloid fibrils, making the biofilm very difficult to degrade by physical and chemical means once formed. By studying the self-assembly mechanism of the fibrils from their monomeric precursors in two unrelated bacteria, our experimental and theoretical approaches shed light on the mechanism of functional amyloid assembly in the context of biofilm formation. Our results suggest that fibril formation may be a rate-limiting step in biofilm formation, which in turn has implications on the protein self-assembly reaction as a target for potential antibiotic drugs.

## INTRODUCTION

A range of microorganisms assemble into large communities, secreting proteins and other molecular species to form a biofilm which allows them to maintain a controlled environment for their growth and proliferation (1–5). In addition to the bacteria themselves, biofilms consist of an extracellular polymeric substance (EPS) that forms the structural scaffold of the biofilm and is composed of polysaccharides, nucleic acids, proteins, and lipids. The EPS provides resistance to physical threats; by enabling adhesion to surfaces, it enables retention of water and forms a physical barrier to some toxins. It also promotes many collaborative effects, e.g., by significantly enhancing the rate of horizontal gene transfer and thus increasing antibiotic resistance (6–9). Aggregated proteins, in the form of functional bacterial amyloids (FuBAs), are a key component of the EPS, providing structural stability to the biofilm (1, 10). Examples of such FuBAs include the *Salmonella* Tafi protein, Xanthomonas axonopodia harpins, Bacillus subtilis TasA protein (1–3), Escherichia coli curli system, and Pseudomonas fluorescens Fap proteins (4, 5).

**FIG 1:**
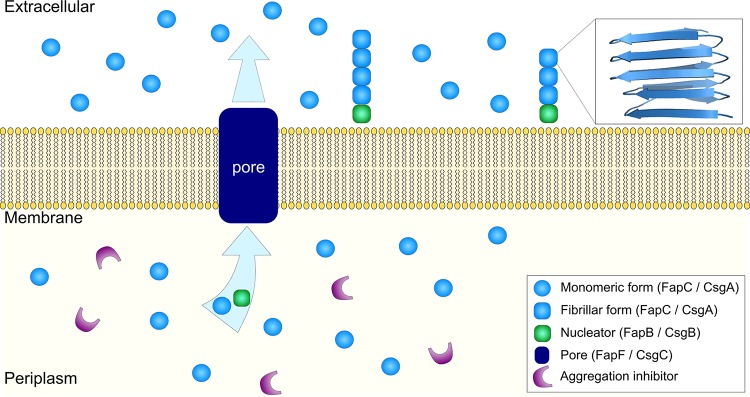
Schematic mechanism of fibril formation for FuBA *in vivo* with the shared components of the functional amyloid systems in E. coli and P. fluorescens. For simplicity, not all components involved in the two functional amyloid systems are included here. The protein (either FapC or CsgA here) is present within the bacterial cells in its monomeric form, alongside a variety of proteins associated with the same operon as the FuBA protein, which are believed to be responsible for inhibiting aggregation. The monomeric protein is then exported to the extracellular space, where nucleator proteins, also part of the same operon, act to initiate aggregation, which then proceeds by the addition of further monomers. The repeat regions of the monomeric proteins comprise the β-strands in the aggregated form (see the predicted structure of CsgA subunits in the aggregated amyloid state on the top right).

Along with the protein that aggregates and forms the main component of the amyloid fibrils in the biofilm, several other support proteins are usually expressed in these FuBA systems. Together they form a system for controlled biofilm formation in the extracellular space, as illustrated in Fig. 1. Generally, to enable biofilm formation and avoid the cytotoxicity commonly associated with unregulated protein aggregation, the amyloidogenic protein needs to be maintained in its soluble form while in the periplasm, exported to the extracellular space, and then nucleated and aggregated on the cell surface. To achieve this goal, aggregation is regulated through transport proteins, transcription factors, chaperones, and even specific auxiliary nucleator proteins that promote the targeted aggregation of monomers at the cell surface (11–15).

Despite the abundance of FuBAs in bacterial habitats, very little is known about the intrinsic molecular mechanism by which they assemble into polymers from their monomeric precursor proteins. In this paper, we present an analysis of the *in vitro* aggregation kinetics of the major amyloid-forming units from the Gram-negative bacteria E. coli curli and P. fluorescens FuBA proteins, namely, CsgA and FapC, in order to study their similarities and differences and examine the results in the context of *in vivo* biofilm formation.

The first protein studied in this work is CsgA, the major component of the amyloid in the form of curli fibrils, in biofilms produced by E. coli. Curli fibrils are the most well-characterized FuBA thus far (4) and have been found to be essential for biofilm formation and bacterial attachment to a wide array of surfaces, ranging from plant cells, to stainless steel, glass, and plastics (16–20). These FuBAs also play key roles in a wide range of interactions with host proteins and in the invasion of host cells (11, 12, 21). The major curli subunit, CsgA, is secreted as an unstructured protein from the cell surface and contains five imperfect repeats with highly conserved glutamine and asparagine residues, considered to be important in amyloid formation (22, 23). Other proteins expressed alongside CsgA are responsible for preventing the aggregation of CsgA within the intercellular environment (CsgC and CsgH [24, 25]) while controlling the initiation of aggregation in the extracellular space through export and promotion of nucleation (CsgG, CsgE, CsgF, and CsgB [12, 23]) (Fig. 1).

The second protein studied in this work is FapC, produced by P. fluorescens. In addition to the original strain studied here, many other *Pseudomonas* strains express Fap amyloids, including the pathologically important Pseudomonas aeruginosa (5, 26, 27). In *Pseudomonas*, the Fap system has been found in proteobacteria where a high fraction of the strains that have been identified are pathogens (39%) or rhizobacteria (36%). The Fap system appears to function as a virulence-enhancing factor in the pathogenic strains.

The major subunit of the amyloid fibrils is the protein FapC (28), which contains three repeat motifs of 37 residues that again include highly conserved glutamine and asparagine residues, separated by highly variable linker regions (5). In analogy to the curli system in E. coli, the additional proteins encoded in the Fap gene are hypothesized to help prevent aggregation within the cell, export FapC to the extracellular space (FapF [29]), or promote nucleation of FapC on the cell surface (5).

## RESULTS

### Structure and morphology.

**FIG 2:**
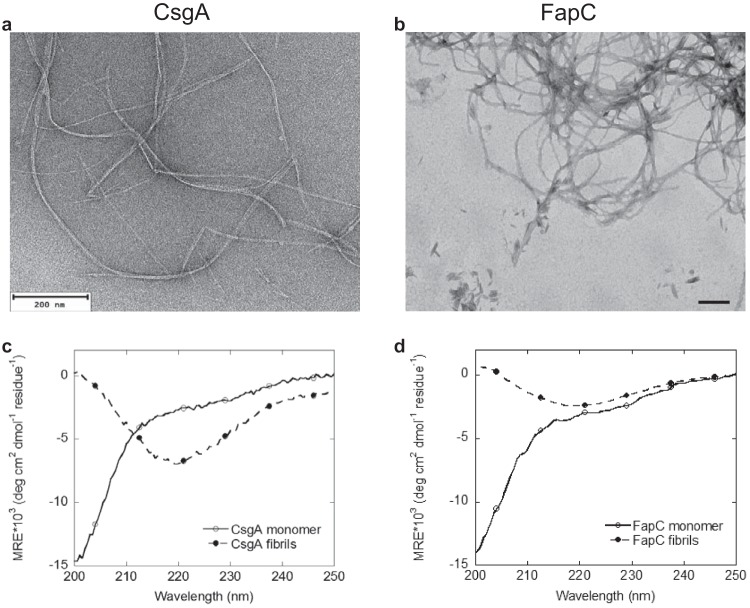
Structural properties of FuBAs. (a) TEM image of fibrils formed by unseeded aggregation of 5 μM of recombinantly expressed and purified CsgA incubated for 5 days at 37°C. Bar, 200 nm. (b) TEM image of fibrils formed by unseeded aggregation of 100 μM of recombinantly expressed and purified FapC incubated for 5 days at 37°C. The fibrils of FapC and CsgA were formed in 20 mM sodium phosphate at pH 7 and 50 mM potassium phosphate at pH 7.4, respectively. Bar, 200 nm. (c) Far-UV CD spectra of monomers and of fibrils of CsgA. (d) Far-UV CD spectra of monomers and of fibrils of FapC. Spectra were recorded under the same conditions as for TEM imaging. For both proteins, the monomeric form displays a CD spectrum with a minimum below 200 nm, indicative of predominantly random coil structure, while the CD spectra of fibrils of both proteins display a single minimum at approximately 218 nm, indicative of β-sheet structure. MRE, mean residue ellipticity.

To assess the properties of FapC amyloids formed *in vitro*, the aggregation of recombinant purified FapC was monitored in solution at pH 7.0 and 37°C. The protein was found to assemble spontaneously to form long, entangled fibrils as observed by transmission electron microscopy (TEM) (Fig. 2b). These findings are similar to those previously observed for the self-assembly of FapC (5, 30) and are consistent with the view that the amyloid-like fibrils formed by FapC are an integral part of the biofilm extracellular matrix.

To assess the changes in secondary structure upon FapC aggregation, we used far-UV circular dichroism (CD) where a transition from a spectrum that has a minimum below 200 nm for the freshly purified monomer to a spectrum with a predominant minimum at approximately 218 nm could be observed after 5 days of incubation at 37°C. This finding is consistent with a transition from a mostly random coil structure to a structure with high β-sheet content upon aggregation (31) (Fig. 2d). Moreover, when monomeric FapC was aggregated in the presence of the amyloid-binding fluorescent dye Thioflavin T (ThT), a significant increase in fluorescence was observed over time, consistent with FapC forming amyloid fibrils.

Similarly, recombinant, purified CsgA monomers were observed to assemble spontaneously into β-sheet-rich fibrillar structures at pH 7.4 and 37°C (Fig. 2a and c). As in the case of FapC, long fibrillar structures could be observed by TEM, while CD measurements again indicate a shift from a spectrum characteristic of a predominantly random coil structure for the freshly purified monomeric protein to one consistent with formation of β-sheet structure upon aggregation following 5 days of incubation at 37°C.

### Kinetic assays.

**FIG 3:**
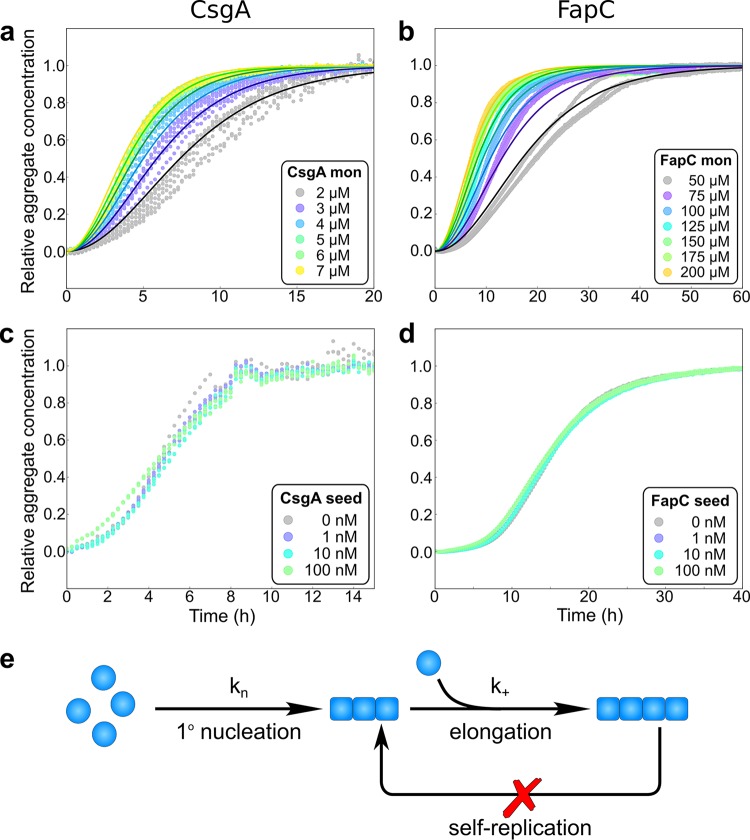
Experimental kinetic data for the aggregation of FapC and CsgA. (a) Aggregation of CsgA from monomeric samples, as measured by ThT fluorescence (colored dots) at 37°C every 15 min under quiescent, i.e., non-shaking, conditions, 50 mM potassium phosphate, pH 7.4. The monomer concentrations were varied between 2 and 7 μM. Six repeats were carried out at each condition. The data are well fit by a nucleation-elongation model (*n_c_* = 1.12, *k_n_k_+_* = 3.68 × 10^4^ M^−nc^ h^−2^, mean residual error [MRE] = 0.00109), schematically shown in panel e, using the AmyloFit interface (33). (b) Aggregation of FapC at 37°C every 10 min, under quiescent conditions, in 20 mM sodium phosphate (pH 7). The monomer concentrations were between 50 and 200 μM, and three repeats were carried out under each condition. The data were fitted by a nucleation-elongation model (*n_c_* = 1.31, *k_n_k_+_* = 1.81 × 10^3^ M^−nc^ h^−2^, MRE = 0.00115). (c) Aggregation of CsgA in the presence and absence of low concentrations of preformed seeds, at a monomer concentration of 5 μM and seed concentrations of 1 to 100 nM monomer equivalents, was carried out in triplicate repeats. No significant effects of added seeds on the rate of aggregation are evident. (d) 100 μM monomeric FapC was aggregated in the absence of preformed fibril seeds and in the presence of seeds at 1, 10, and 100 nM monomer equivalents in triplicate repeats. No significant effects on the rate of aggregation are evident. (e) Schematic illustration of a nucleation-elongation mechanism for fibrillar assembly. Monomers nucleate with rate constant *k_n_* and reaction order *n_c_*; the nuclei then grow by the addition of further monomers to the fibril ends with rate constant *k_+_*.

To acquire a detailed molecular understanding of the mechanism by which the monomeric proteins convert into fibrillar structures, we conducted an analysis of the aggregation kinetics for both FapC and CsgA at a range of monomer concentrations (Fig. 3). A comprehensive set of kinetic models of protein aggregation (32, 33) based on the fundamental steps of self-assembly has successfully been applied to describe many phenomena in protein aggregation in recent years (34–36). Through these models, the kinetics of aggregation provide access to the rates and reaction orders of the underlying molecular processes, thus for example, allowing one to determine whether new aggregates are mainly formed by the self-replication of existing aggregates or directly from monomers via nucleation. Here, aggregation kinetics were obtained by monitoring the fluorescence intensity of ThT, which is increased upon binding to amyloid fibrils, during the aggregation from initially monomeric protein (37). For both proteins, the ThT fluorescence intensities at the end points of the aggregation reaction were found to scale linearly with the protein concentration, as shown in Fig. S1 in the supplemental material, indicating that the signal intensity is proportional to the quantity of fibrils formed. Both proteins exhibited reproducible sigmoidal aggregation curves without any pronounced lag phase. However, while the aggregation of CsgA was essentially complete within 10 to 20 h at monomer concentrations between 2 and 7 μM, the aggregation of FapC was significantly slower, taking 20 to 40 h to reach completion, even at considerably higher monomer concentrations between 50 and 200 μM (Fig. 3a and b).

10.1128/mBio.02279-18.1 FIG S1 CD analysis of FapC and CsgA monomers and end-ThT scaling with initial monomer concentration and half-time analysis. (Top) CD analysis of FapC and CsgA monomer structure. (A) Far-UV CD spectra of FapC and CsgA monomers. (B) Deconvolution of the Far-UV CD spectra shown in panel A using the K2d algorithm on the Dichroweb server (1, 2). The percentage contribution of various structural components is given for the monomeric form of both proteins. (Middle) A linear scaling of the ThT fluorescence intensity with respect to the monomer protein concentration is seen for both proteins. The error bars are shown in the plot. (C) End-ThT level of FapC triplicates plotted against the initial monomer concentration. (D) End-ThT level of six replicates of CsgA plotted against the initial monomer concentration. (Bottom) The logarithm of the half time (the time it takes to reach half of the maximum ThT signal) of aggregation plotted against the logarithm of the initial monomer concentration. (E) Half-time analysis of FapC kinetic data gives a straight line with the slope giving a scaling component of −0.665. (F) Half-time analysis of CsgA kinetic data gives a straight line with the slope giving a scaling component of −0.563. The straight lines in both plots indicate that the dominant mechanism of fibril multiplication is the same for all monomer concentrations for both proteins. Download FIG S1, PDF file, 0.3 MB. Copyright © 2019 Andreasen et al. 2019 Andreasen et al. This content is distributed under the terms of the Creative Commons Attribution 4.0 International license.

**FIG 4:**
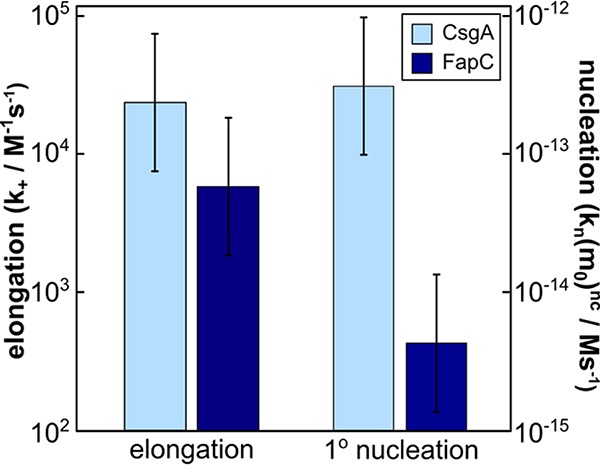
Comparison of the rate constants of primary nucleation and the elongation for CsgA and FapC. The rate constants of elongation (left bars, left axis), are comparable for the two proteins. The rate of primary nucleation evaluated at monomer concentrations of 10 μM (right bars, right axis), is significantly higher for CsgA than for FapC, which accounts for the significant differences in overall aggregation rate of the two proteins. The error bars denote a factor of three, a conservative estimate of the error which originates mainly from the inaccuracy in the determination of the fibril dimensions (see Materials and Methods).

In order to determine the mechanism of aggregation, the data for each protein system were fitted globally, at all monomer concentrations simultaneously, by kinetic equations using the Amylofit interface (33) (see Text S1 in the supplemental material for details on the equations used in the fitting). High-quality global fits were achieved for both proteins assuming a simple nucleated polymerization (or nucleation-elongation) mechanism (Fig. 3e). In this model of linear self-assembly, the protein monomers form an initial nucleus with rate constant *k_n_* and reaction order *n_c_*. In subsequent steps, the aggregates then grow by the addition of further monomers to the fibril ends with rate constant *k_+_*. Notably, each new fibril must be initiated through a specific nucleation event from monomeric proteins in this mechanism. Of particular importance is the observation that, to be consistent with this nucleated polymerization mechanism, the fibrils themselves are unable to self-replicate through secondary processes to any significant degree. This result was further verified through seeded experiments as detailed below. This observed lack of self-replication is in contrast to some disease-related amyloid fibrils, such as those formed by the Aβ peptide (34) or α-synuclein (38) protein, associated with Alzheimer’s and Parkinson’s disease, respectively (39, 40), where the main generator of new aggregates is a surface catalyzed self-replication process. Thus, a negligible rate of self-replication is potentially a requirement for the controlled formation of biofilm structures. The kinetic parameters for the global fits for FapC and CsgA are shown in Fig. 4 and Table S1 in the supplemental material.

10.1128/mBio.02279-18.5 TABLE S1 Kinetic parameters for global fitting of the experimental data for FapC and CsgA to a nucleation-elongation model, where *n_c_* is the reaction order of the primary nucleation process, *k_n_* is the rate constant for the primary nucleation process, and *k*_+_ is the rate constant for the elongation of existing fibrils. Download Table S1, PDF file, 0.1 MB. Copyright © 2019 Andreasen et al. 2019 Andreasen et al. This content is distributed under the terms of the Creative Commons Attribution 4.0 International license.

10.1128/mBio.02279-18.8 TEXT S1 Equations for global fitting to a nucleation-elongation model. Download Text S1, PDF file, 0.2 MB. Copyright © 2019 Andreasen et al. 2019 Andreasen et al. This content is distributed under the terms of the Creative Commons Attribution 4.0 International license.

To probe further the mechanism of aggregation through nucleated polymerization, and in particular to verify the absence of any significant self-replication processes, we performed experiments in the presence of low concentrations of preformed fibril seeds (Fig. 3c and d). Such experiments provide a direct means of probing the ability of fibrils to self-replicate (41, 42). When up to 1% of the total protein mass present at the start of the reaction was in the form of seed fibrils, no effects on the aggregation kinetics of either CsgA or FapC were observed, confirming the absence of detectable levels of self-replication processes under the conditions studied and hence supporting the proposed nucleation-elongation mechanism. Finally, to explore the relevance of this kinetic analysis performed in solution *in vitro* to processes occurring at the surfaces of bacterial cells, we derived the integrated rate laws describing the kinetics of the aggregation process taking place at a surface (Text S2). We find that, as long as monomeric proteins diffuse faster than they are being consumed by aggregates, the model recovers the same form as that used for the analysis of the *in vitro* data.

10.1128/mBio.02279-18.9 TEXT S2 Integrated rate law for kinetics of aggregation at the cell surface. Download Text S2, PDF file, 0.2 MB. Copyright © 2019 Andreasen et al. 2019 Andreasen et al. This content is distributed under the terms of the Creative Commons Attribution 4.0 International license.

### Origin of the differences in FapC and CsgA aggregation.

The global fits to the time courses of the aggregation of FapC or CsgA from monomeric protein solutions yield a combined elongation-nucleation rate constant *k_n_k_+_* (Table S1). The value of this combined rate constant was found to be approximately 3 orders of magnitude higher for CsgA than for FapC. In agreement with this finding, the overall aggregation propensity score assigned to each protein by means of the prediction algorithm Zyggregator (43, 44) is significantly higher for CsgA (0.93 ± 0.05 compared to 0.80 ± 0.04 for FapC [Fig. S2]). The increased number of repeat units in CsgA, five compared to the three found for FapC, may be a reason for its elevated aggregation propensity compared to FapC.

10.1128/mBio.02279-18.2 FIG S2 The aggregation propensity at each amino acid position for CsgA and FapC as calculated by the aggregation propensity prediction algorithm Zyggregator (4). Using the software, the overall aggregation propensity, *z*_agg_, of FapC is found to be 0.801 ± 0.039, while for CsgA, it is 0.928 ± 0.046. Download FIG S2, PDF file, 0.2 MB. Copyright © 2019 Andreasen et al. 2019 Andreasen et al. This content is distributed under the terms of the Creative Commons Attribution 4.0 International license.

In order to determine the relative contributions of the rates of elongation and nucleation to the difference in the overall aggregation rate, we carried out aggregation experiments in the presence of high concentrations of preformed fibril seeds. These experiments, together with measurement of the average length of seed fibrils (Fig. S3 and S4 and Tables S2 and S3), allow the rate constant of elongation to be estimated in each case. The values of the elongation rate constants determined for the two proteins were found to be similar, differing by only a factor of 4, which is insignificant given the uncertainties inherent in the estimation of the fibril seed lengths. This finding indicates that the major contribution to the differences in the *in vitro* aggregation rates between the two proteins is the significantly lower primary nucleation rate of FapC (Fig. 4).

10.1128/mBio.02279-18.3 FIG S3 Fitting of seeded aggregation kinetic data of FapC. (Top) Analysis of the initial gradient during seeded aggregation used to estimate the elongation rates. The initial gradient (first 30 min) of the above two data sets is plotted against the free monomer concentration. A straight line was fitted to these points, its slope was proportional to the number of seed fibrils, and the elongation rate was constant. A slight effect of saturation of elongation can be seen at high concentrations, as observed frequently in such systems (6–8); hence, these points (red) were excluded. (Bottom) Global fitting of seeded aggregation kinetic data. The data were fitted to a nucleation elongation model with added seeds. The parameters *k*_+_ and *k_n_* were allowed to vary, while their product *k*_+_ *k_*n*_* was held constant at the value obtained for the unseeded data (see Table S2). For FapC, a constant seed concentration of 50 μM and monomer concentrations ranging from 25 μM to 175 μM are used. For CsgA, a constant seed concentration of 5.3 μM and monomer concentrations ranging from 2.72 μM to 9.08 μM are used. Download FIG S3, PDF file, 0.2 MB. Copyright © 2019 Andreasen et al. 2019 Andreasen et al. This content is distributed under the terms of the Creative Commons Attribution 4.0 International license.

10.1128/mBio.02279-18.4 FIG S4 Measurements of fibril seed lengths from TEM image of sonicated fibril seeds. The scale bar indicates 100 nm. The image is of FapC fibril seeds and is representative of a larger set of fibril seeds analyzed. Download FIG S4, PDF file, 0.2 MB. Copyright © 2019 Andreasen et al. 2019 Andreasen et al. This content is distributed under the terms of the Creative Commons Attribution 4.0 International license.

10.1128/mBio.02279-18.6 TABLE S2 Kinetic parameters for global fitting of the experimental data for seeded aggregation of FapC and CsgA to a nucleation-elongation model. *M*_0_ is the concentration of fibril seeds. *P*_0_ is the number concentration of seed fibrils. *n_c_* is the reaction order of the primary nucleation process. *k_n_* is the rate constant for the primary nucleation process, and *k*_+_ is the rate constant for the elongation of existing fibrils. The values for the elongation rates are within a factor of two of those determined from the initial gradient analysis. Download Table S2, PDF file, 0.1 MB. Copyright © 2019 Andreasen et al. 2019 Andreasen et al. This content is distributed under the terms of the Creative Commons Attribution 4.0 International license.

10.1128/mBio.02279-18.7 TABLE S3 Dimensions of FapC and CsgA fibril seeds in nanometers extracted from TEM images. A total of 480 fibril seeds for both proteins have been analyzed. An average density of amino acids of 1.35 g/cm^3^ is used in the calculations. Download Table S3, PDF file, 0.1 MB. Copyright © 2019 Andreasen et al. 2019 Andreasen et al. This content is distributed under the terms of the Creative Commons Attribution 4.0 International license.

As *in vivo* the aggregation process of both CsgA and FapC is thought to be actively initiated on the cell surface, the intrinsic primary nucleation rates we observed *in vitro* are unlikely to be relevant in the *in vivo* context. In contrast, the *in vitro* elongation rates are likely to be good estimates of the *in vivo* rates, a conclusion that is consistent with the fact that the biofilms of the two bacterial species are formed over comparable timescales *in vivo* (45, 46). Since both proteins originate from Gram-negative species, this could indicate a general mechanism for Gram-negative bacterial species forming biofilms.

### Comparison of the rates of aggregation and biofilm growth.

**FIG 5:**
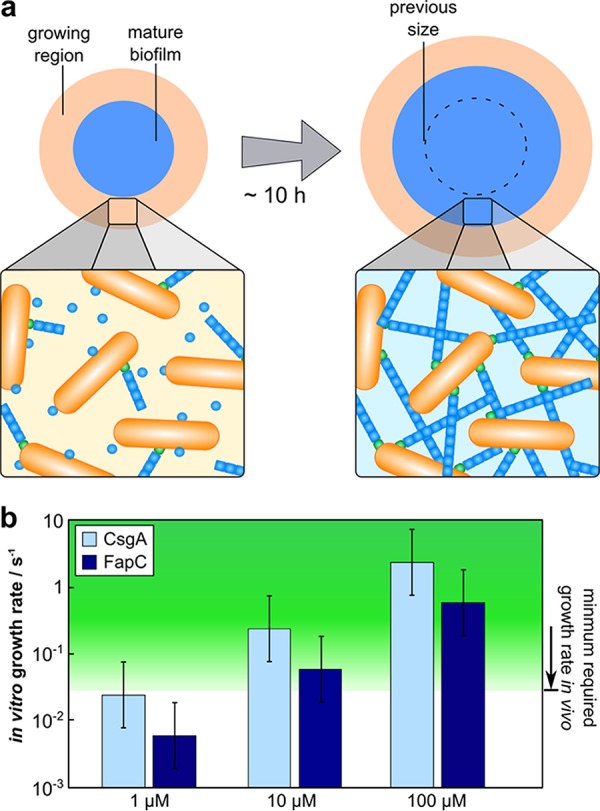
Comparison of *in vitro* and *in vivo* aggregation. (a) The formation of the biofilm matrix occurs in a region that is located along the edge of the biofilm (orange). Cells spend approximately 10 h in this region until the biofilm is mature (blue). During this time, an interlinked network of amyloid fibrils is formed. (b) The amyloid growth rates predicted from the *in vitro* measurements of the elongation rate constant at 1, 10, and 100 μM monomeric protein (blue bars). The green region highlights the fibril growth rate required to allow for the observed spreading rates of the biofilms *in vivo*.

In order to explore the significance of the values of the elongation rates obtained here, we have compared them to the speed of biofilm spreading *in vivo* measured previously. While our *in vitro* measurements lack several components found in the EPS, such as polysaccharides, nucleic acids, and lipids, they allow us to measure the intrinsic rate of amyloid growth, a subprocess of the complex mechanism of biofilm formation. The rate *in vivo* may be limited by slow diffusion in the EPS or competition of other species with free fibril ends and monomers or may be increased by the presence of interfaces for fibrils to grow on. Regardless of the specific mechanism, from measurements of the total timescale of biofilm formation, we can obtain a lower bound on the rate of amyloid formation required, as the subprocess of amyloid formation must be completed within the time of overall biofilm formation. Studies of biofilm formation *in vivo* have revealed that the biofilm EPS matrix is produced predominantly at the edge of the biofilm by cells in a region of constant thickness, which spreads outwards as the central regions of the biofilm mature (Fig. 5). By using previously published measurements of the thickness and spreading speed of this region, we find that an amyloid growth rate on the order of 100 nm/h or 0.03 nm/s is required to allow for the observed rates of biofilm formation, the estimation is detailed in Text S3 in the supplemental material. Note that this is a lower bound, so the actual rate of amyloid formation *in vivo* may be significantly higher, but it cannot be lower if a biofilm is to be formed on the observed timescales.

Having thus obtained a requirement for the minimal rate of amyloid growth from direct observations of biofilm formation *in vivo*, we now compare this rate to our *in vitro* measurements. To convert the *k_+_* rate constant to a growth rate in units of length per time, the concentration of available monomer, *m*_0_, needs to be estimated, as the rates are determined by the product of the rate constant and monomer concentration, *k_+_m*_0_.

The rates of growth from the *in vitro* measurements for a number of different monomer concentrations are shown in Fig. 5b and compared to the lower bound for the rate estimated *in vivo*. Strikingly, the growth rates that we measured *in vitro* are low compared to the minimal required rate *in vivo*. Only at monomer concentrations of tens of micromolar are the required growth rates of 0.03 nm/s reached. Based on our observations of the total yield of FuBAs in bacterial cultures, the extracellular monomer concentration in the biofilm is likely to be below 10 μM (Text S3). Thus, there must be mechanisms in the biofilm that lead to an increased rate of amyloid growth, either by increasing the local concentration of monomeric precursors or by other means such as a surface to template growth (for example, Sleutel et al. [47] find a significantly higher rate of growth on mica surfaces than we do in solution). This low rate of aggregation compared to the speed of biofilm spread suggests both a potential role of amyloid formation as a control mechanism in biofilm formation and a desire by the organism to minimize the aggregation propensity.

10.1128/mBio.02279-18.10 TEXT S3 Details of comparison to *in vivo* behavior. Download Text S3, PDF file, 0.3 MB. Copyright © 2019 Andreasen et al. 2019 Andreasen et al. This content is distributed under the terms of the Creative Commons Attribution 4.0 International license.

## DISCUSSION

We have conducted a detailed kinetic analysis of the aggregation of two functional amyloid proteins from unrelated bacteria, E. coli and P. fluorescens. We found that both proteins aggregate through a mechanism involving only primary nucleation and elongation, while the contribution of secondary processes that give rise to self-replication of aggregates is negligible in both cases. *In vivo*, the fact that the fibrils do not self-replicate may allow the generation of new fibrils to be initiated exclusively through nucleator proteins at the cell surface. By preventing fibrils from self-replicating at random locations in the extracellular space, this mechanism thus provides the bacterial cells with the possibility to regulate both the number and location of the fibrils formed, and thereby to control important properties of the biofilm.

It is remarkable that even though the two proteins from E. coli and P. fluorescens are genetically distinct (with only approximately 10% sequence identity), they show striking similarities in their aggregation behavior. The differences observed during *in vitro* aggregation are almost exclusively due to differences in the rates of nucleation, a process that is likely to proceed *in vivo* via a different pathway, controlled by additional nucleator proteins. In contrast, the rate constants of fibril growth are very similar for the two proteins and significantly lower than those observed for disease-related amyloid fibrils such as those of the Aβ polypeptide (35).

By comparing the rates of biofilm growth *in vivo* to those of amyloid elongation *in vitro*, we found that to achieve the observed rates of biofilm spreading, high local concentrations of the monomeric precursor proteins or other rate-enhancing factors are required *in vivo*. Thus, it appears that these functional amyloid structures have been optimized to strike a balance between a relatively low aggregation propensity, potentially to avoid unwanted aggregation within the cell, and fast biofilm formation that is required in a functional context. The likely presence of specific mechanisms to locally concentrate monomeric protein molecules or even to enhance the rate of elongation in relevant locations of the biofilm by other means suggests that amyloid growth may be a rate-limiting step in biofilm formation. If this were the case, biofilm growth could be inhibited by inhibitors of amyloid formation. Indeed, there is evidence that inhibitors of aggregation also tend to inhibit biofilm formation (48, 49). In the context of Parkinson’s and Alzheimer’s disease, the inhibition of amyloid formation is a major therapeutic target, with the inhibition of self-replication by secondary nucleation being the most promising candidate (36). We propose that similarly, biofilm formation may be targeted by aggregation inhibitors, but unlike in the aggregation of Aβ and α-synuclein in Alzheimer’s and Parkinson’s disease, in the case of biofilm-forming amyloids, one should target the elongation step rather than the self-replication step.

In conclusion, nature appears to favor proteins with a specific set of self-assembly properties as the building blocks for the amyloid components of biofilms. Namely, such proteins should display a low elongation rate (possibly to provide a means of limiting biofilm formation) and lack the ability to self-replicate to allow control over the rate of formation and over the location of new fibrils. We believe the key processes and mechanisms revealed in this work will set the ground for understanding the role of amyloid growth as a potentially limiting factor of biofilm formation and highlight that growth rather than self-replication of fibrils should be the target for potential inhibitory agents.

## MATERIALS AND METHODS

His-tagged FapC and CsgA were recombinantly expressed without the corresponding signal sequence using Escherichia coli BL21(DE3). Both proteins were purified using Ni-affinity chromatography resin in 8 M guanidine hydrochloride buffer. FapC was desalted into 20 mM sodium phosphate (pH 7) using a PD10 desalting column (GE Healthcare, USA). CsgA was desalted into 50 mM potassium phosphate (pH 7.4) using a HiTrap desalting column (GE Healthcare, USA). The aggregation kinetics of both proteins were monitored during incubation at 37°C using ThT fluorescence. For seeded experiments, the preformed and sonicated fibril seeds were added immediately before incubation. The kinetics of aggregation were fitted globally using the AmyloFit interface at www.amylofit.ch.cam.ac.uk.

### Recombinant expression and purification of FapC.

E. coli BL21(DE3) cells were transformed with the pET 28a vector containing the gene for FapC (residues 25 to 250) from the Pseudomonas fluorescens strain UK4 without the signal sequence (residue 1 to 24) and with the six-residue His tag at the C terminus. The cells were grown on LB agar plates with kanamycin at 37°C, and the colonies were transferred to LB medium with kanamycin and grown to an optical density at 600 nm (OD_600_) of ∼1. Protein expression was induced by adding IPTG to a final concentration of 1 mM, followed by incubation for 3 h. The cells were harvested and resuspended in 20 ml 50 mM Tris-HCl (pH 8) and 8 M guanidine hydrochloride per liter of culture and lysed by sonication. Cell debris was removed by centrifugation for 30 min at 5,000 × *g*. The supernatant was then loaded onto a His-trap column (Super Nickel NTA resin; Generon, UK). The column was washed with increasing concentrations of imidazole (0, 30, 60, and 120 mM in 50 mM Tris-HCl [pH 8], 8 M guanidine hydrochloride) and eluted with 300 mM imidazole, 50 mM Tris-HCl (pH 8), and 8 M guanidine hydrochloride. The resulting fractions were analyzed by SDS-PAGE following ethanol precipitation, and the guanidine hydrochloride was removed before use, using a PD10 desalting column (GE Healthcare, USA) equilibrated in 20 mM sodium phosphate (pH 7) using the gravity protocol.

### Recombinant expression and purification of CsgA.

The gene encoding the mature form of CsgA (residues 22 to 151) was amplified by PCR from E. coli BL21(DE3) genomic DNA. The product was ligated into pET28a using NcoI and XhoI sites to be in frame with a C-terminal six-residue His tag. CsgA_22-151_-His was expressed in *E*. *coli* BL21(DE3) cells grown in TB medium at 37°C. The cells were grown to an OD_600_ of ∼1.0, and protein expression was induced by adding IPTG to a final concentration of 0.5 mM. After 1.5 h, the cells were harvested by centrifugation at 4,000 × *g* in 300-ml aliquots and flash frozen in liquid nitrogen. Purification of CsgA was performed essentially as described by Zhou et al. (50), with modifications as described below. Each aliquot of frozen cells was defrosted and mixed thoroughly in 30 ml solubilization buffer (8 M guanidine hydrochloride, 50 mM potassium phosphate, 0.1 M NaCl [pH 7.8]). The samples were then sonicated to promote cell lysis and to break up any preformed CsgA aggregates using a 4-mm probe tip for 60 s in 0.5-s bursts. After gentle rocking at room temperature for 1 to 2 h, the lysate was centrifuged at 17,000 rpm to remove insoluble debris. Solubilized CsgA was captured by adding 600 µl TALON resin (TaKaRa Bio, USA) in a 50% slurry and rocking gently for 1 h at room temperature. The resin was collected by centrifugation for 3 min at 500 × *g* and transferred to a 1-ml polypropylene column (Qiagen, Netherlands) and moved to a cold room for subsequent steps. Nonspecifically bound protein was washed off the column using sequential aliquots of the following: (i) 1 ml solubilization buffer; (ii) 1 ml ice-cold 1.6 M guanidine hydrochloride, 50 mM potassium phosphate, 0.1 M NaCl (pH 7.8); (iii) 1 ml ice-cold 50 mM potassium phosphate, 0.1 M NaCl (pH 7.8); (iv) 2 ml ice-cold 50 mM potassium phosphate, 0.1 M NaCl, 2 mM imidazole (pH 7.8). CsgA was eluted directly into a 4-ml Amicon centrifugal concentrator (30,000 MWCO, prewashed in assay buffer) using 1.6 ml ice-cold 50 mM potassium phosphate, 0.1 M NaCl, 500 mM imidazole (pH 7.8). The washing and elution steps were performed in less than 5 min to minimize fibril formation prior to use. The sample was centrifuged for 10 min at 4,000 × *g* at 4°C to remove aggregates, and the filtrate was injected through a 5-ml HiTrap desalting column (GE Healthcare, USA) preequilibrated in ice-cold assay buffer (50 mM potassium phosphate, pH 7.4). Protein concentrations were measured using a Nanodrop spectrophotometer (Thermo Scientific, USA).

### Measurement of FapC aggregation kinetics.

Samples of desalted FapC were passed through a 0.22-μm filter and diluted to the required concentrations. ThT was added to the protein solutions to a final concentration of 40 μM, and the solutions were transferred to 96-well black Corning polystyrene half-area microtiter plates with a nonbinding surface. The plates were sealed to prevent evaporation and placed in a Fluostar Omega plate reader (BMG Labtech, Germany). The plates were incubated at 37°C under quiescent conditions, and the ThT fluorescence (excitation, 450 nm; emission, 482 nm) was measured every 10 min. When experiments were carried out in the presence of preformed fibril seeds, these were added immediately before the plates were sealed. Fibril seeds were produced by sonicating protein fibrils that had been generated by incubation of 100 μM FapC at 37°C under quiescent conditions for 4 days. The protein fibrils were sonicated for 30 s using a Sonoplus sonication probe (Bandelin, Germany). Note that the lower reproducibility of aggregation kinetics at low concentrations is likely a consequence of extrinsic stochastic factors that have an increased effect at low protein concentrations over the long aggregation timescales.

### Measurement of CsgA aggregation kinetics.

Samples of desalted CsgA were diluted into 50 mM potassium phosphate buffer at pH 7.4 as required. ThT was added to the protein solutions to a final concentration of 40 μM, and the solutions were transferred to 96-well black Corning polystyrene half-area microtiter plates with a nonbinding surface. The plates were sealed to prevent evaporation and placed in a SpectraMax M2e plate reader (Molecular Devices, USA). The plates were incubated at 37°C, and the ThT fluorescence (excitation, 438 nm; emission, 495 nm) was measured every 15 min with shaking for 10 s prior to each measurement. When fibril seeds were present, they were added immediately before the plate was sealed. The fibril seeds were produced by sonicating protein fibrils for 30 s using a Sonoplus sonication probe (Bandelin, Germany). The kinetics of aggregation were fitted globally using the AmyloFit interface (33) at www.amylofit.ch.cam.ac.uk. The equations used in the fitting are based on a master equation approach (32) and are given in Text S1 in the supplemental material.

### Transmission electron microscopy (TEM).

Fibril samples (5 μl) were applied to carbon-coated nickel grids, stained with 2% (wt/vol) uranyl acetate, and imaged on a FEI Tecnai G_2_ transmission electron microscope (Multi-Imaging Unit in the Department of Physiology, Development and Neuroscience, University of Cambridge, UK). Images were analyzed using the SIS Megaview II Image Capture system (Olympus, Japan).

### Calculation of the elongation rate constant.

Seeded aggregation with high concentrations of preformed fibril seeds was carried out at 5.3 μM fibrils and monomer concentrations between 2 and 9 μM for CsgA and 50 μM fibrils at monomer concentrations between 50 and 200 μM for FapC. These experiments were used to estimate the rates of fibril elongation. The initial gradients (first 30 min) of the kinetic curves were determined and plotted against the monomer concentrations (Fig. S3). The increases in the initial rates were found to be 0.6 h^−1^ for FapC and 0.7 h^−1^ for CsgA. This quantity corresponds to *k*_+_*P*_0_, where *k*_+_ is the elongation rate constant and *P*_0_ is the number concentration of seed fibrils (for details, see references 33 and 35). The *P*_0_ values were determined from measurements of the average seed length from TEM (Fig. S4) and the knowledge of the mass concentration of seeds. The average seed lengths were found to be 30 nM for FapC and 8 nM for CsgA and were estimated to be accurate to within a factor of approximately three (standard errors of the measurements are significantly lower, but we give this more generous estimate of the error to account for the fact that the distributions measured by TEM may be biased from the distributions actually present in solution during seeding). This information enabled us to give a final estimate of the rate constants of elongation as approximately 6,000 s^−1^ M^−1^ for FapC and 24,000 s^−1^ M^−1^ for CsgA. Given the uncertainties in the estimations of *P*_0_ and the initial gradients, these two rate constants do not differ significantly in comparison to differences in other kinetic parameters. For consistency, the seeded aggregation experiments were also fitted to the same kinetic equations as the unseeded ones, yielding good fits and elongation rates of 3,100 s^−1^ M^−1^ for FapC and 21,000 s^−1^ M^−1^ for CsgA, in close agreement with the values from measurements of the initial gradients (Fig. S3).

### Far-UV circular dichroism spectroscopy.

Circular dichroism (CD) spectra for wavelengths from 250 nm to 200 nm, with a step size of 0.5 nm, bandwidth of 2 nm, and scan speed of 50 nm/min, were recorded at 25°C with a J-810 CD spectrometer (Jasco, Japan) using a 1-mm quartz cuvette (Hellma, Germany). Five spectra were averaged for each sample, and the buffer spectra were subtracted from each. Samples containing fibrils were subjected to sonication for 2 s with a Sonoplus sonication probe (Bandelin, Germany) and inspected to ensure the absence of visible aggregates in the cuvette prior to analysis.

## References

[1] Romero D, Aguilar C, Losick R, Kolter R 2010 Amyloid fibers provide structural integrity to Bacillus subtilis biofilms. Proc Natl Acad Sci U S A 107:2230–2234. doi:10.1073/pnas.0910560107. 20080671 PMC2836674

[2] Oh J, Kim JG, Jeon E, Yoo CH, Moon JS, Rhee S, Hwang I 2007 Amyloidogenesis of type III-dependent harpins from plant pathogenic bacteria. J Biol Chem 282:13601–13609. doi:10.1074/jbc.M602576200. 17314101

[3] Collinson SK, Emody L, Muller KH, Trust TJ, Kay WW 1991 Purification and characterization of thin, aggregative fimbriae from Salmonella enteritidis. J Bacteriol 173:4773–4781. 1677357 10.1128/jb.173.15.4773-4781.1991 PMC208156

[4] Chapman MR, Robinson LS, Pinkner JS, Roth R, Heuser J, Hammar M, Normark S, Hultgren SJ 2002 Role of Escherichia coli curli operons in directing amyloid fiber formation. Science 295:851–855. doi:10.1126/science.1067484. 11823641 PMC2838482

[5] Dueholm MS, Petersen SV, Sonderkaer M, Larsen P, Christiansen G, Hein KL, Enghild JJ, Nielsen JL, Nielsen KL, Nielsen PH, Otzen DE 2010 Functional amyloid in Pseudomonas. Mol Microbiol 77:1009–1020. doi:10.1111/j.1365-2958.2010.07269.x. 20572935

[6] Prosser BL, Taylor D, Dix BA, Cleeland R 1987 Method of evaluating effects of antibiotics on bacterial biofilm. Antimicrob Agents Chemother 31:1502–1506. 3435100 10.1128/aac.31.10.1502 PMC174979

[7] Nickel JC, Ruseska I, Wright JB, Costerton JW 1985 Tobramycin resistance of Pseudomonas aeruginosa cells growing as a biofilm on urinary catheter material. Antimicrob Agents Chemother 27:619–624. 3923925 10.1128/aac.27.4.619 PMC180108

[8] Mah TF, O'Toole GA 2001 Mechanisms of biofilm resistance to antimicrobial agents. Trends Microbiol 9:34–39. 11166241 10.1016/s0966-842x(00)01913-2

[9] Flemming HC, Wingender J 2010 The biofilm matrix. Nat Rev Microbiol 8:623–633. doi:10.1038/nrmicro2415. 20676145

[10] Fowler DM, Koulov AV, Balch WE, Kelly JW 2007 Functional amyloid–from bacteria to humans. Trends Biochem Sci 32:217–224. doi:10.1016/j.tibs.2007.03.003. 17412596

[11] Gebbink MF, Claessen D, Bouma B, Dijkhuizen L, Wosten HA 2005 Amyloids–a functional coat for microorganisms. Nat Rev Microbiol 3:333–341. doi:10.1038/nrmicro1127. 15806095

[12] Barnhart MM, Chapman MR 2006 Curli biogenesis and function. Annu Rev Microbiol 60:131–147. doi:10.1146/annurev.micro.60.080805.142106. 16704339 PMC2838481

[13] Epstein EA, Chapman MR 2008 Polymerizing the fibre between bacteria and host cells: the biogenesis of functional amyloid fibres. Cell Microbiol 10:1413–1420. doi:10.1111/j.1462-5822.2008.01148.x. 18373633 PMC2674401

[14] Otzen DE 2011 Assembling good amyloid: some structures at last. Structure 19:1207–1209. doi:10.1016/j.str.2011.08.005. 21893282

[15] Taylor JD, Zhou Y, Salgado PS, Patwardhan A, McGuffie M, Pape T, Grabe G, Ashman E, Constable SC, Simpson PJ, Lee WC, Cota E, Chapman MR, Matthews SJ 2011 Atomic resolution insights into curli fiber biogenesis. Structure 19:1307–1316. doi:10.1016/j.str.2011.05.015. 21893289 PMC3173608

[16] Jeter C, Matthysse AG 2005 Characterization of the binding of diarrheagenic strains of E. coli to plant surfaces and the role of curli in the interaction of the bacteria with alfalfa sprouts. Mol Plant Microbe Interact 18:1235–1242. doi:10.1094/MPMI-18-1235. 16353558

[17] Ryu JH, Beuchat LR 2005 Biofilm formation by Escherichia coli O157:H7 on stainless steel: effect of exopolysaccharide and Curli production on its resistance to chlorine. Appl Environ Microbiol 71:247–254. doi:10.1128/AEM.71.1.247-254.2005. 15640194 PMC544232

[18] Ryu JH, Kim H, Frank JF, Beuchat LR 2004 Attachment and biofilm formation on stainless steel by Escherichia coli O157:H7 as affected by curli production. Lett Appl Microbiol 39:359–362. doi:10.1111/j.1472-765X.2004.01591.x. 15355539

[19] Prigent-Combaret C, Prensier G, Le Thi TT, Vidal O, Lejeune P, Dorel C 2000 Developmental pathway for biofilm formation in curli-producing Escherichia coli strains: role of flagella, curli and colanic acid. Environ Microbiol 2:450–464. 11234933 10.1046/j.1462-2920.2000.00128.x

[20] Otzen D, Nielsen PH 2008 We find them here, we find them there: functional bacterial amyloid. Cell Mol Life Sci 65:910–927. doi:10.1007/s00018-007-7404-4. 18034321 PMC11131872

[21] Kanamaru S, Kurazono H, Terai A, Monden K, Kumon H, Mizunoe Y, Ogawa O, Yamamoto S 2006 Increased biofilm formation in Escherichia coli isolated from acute prostatitis. Int J Antimicrob Agents 28(Suppl 1):S21–S25. doi:10.1016/j.ijantimicag.2006.05.006. 16828264

[22] Wang X, Chapman MR 2008 Sequence determinants of bacterial amyloid formation. J Mol Biol 380:570–580. doi:10.1016/j.jmb.2008.05.019. 18565345 PMC2478699

[23] Andersson EK, Bengtsson C, Evans ML, Chorell E, Sellstedt M, Lindgren AE, Hufnagel DA, Bhattacharya M, Tessier PM, Wittung-Stafshede P, Almqvist F, Chapman MR 2013 Modulation of curli assembly and pellicle biofilm formation by chemical and protein chaperones. Chem Biol 20:1245–1254. doi:10.1016/j.chembiol.2013.07.017. 24035282 PMC4243843

[24] Taylor JD, Hawthorne WJ, Lo J, Dear A, Jain N, Meisl G, Andreasen M, Fletcher C, Koch M, Darvill N, Scull N, Escalera-Maurer A, Sefer L, Wenman R, Lambert S, Jean J, Xu Y, Turner B, Kazarian SG, Chapman MR, Bubeck D, de Simone A, Knowles TP, Matthews SJ 2016 Electrostatically-guided inhibition of Curli amyloid nucleation by the CsgC-like family of chaperones. Sci Rep 6:24656. doi:10.1038/srep24656. 27098162 PMC4838910

[25] Evans ML, Chorell E, Taylor JD, Aden J, Gotheson A, Li F, Koch M, Sefer L, Matthews SJ, Wittung-Stafshede P, Almqvist F, Chapman MR 2015 The bacterial curli system possesses a potent and selective inhibitor of amyloid formation. Mol Cell 57:445–455. doi:10.1016/j.molcel.2014.12.025. 25620560 PMC4320674

[26] Dueholm MS, Sondergaard MT, Nilsson M, Christiansen G, Stensballe A, Overgaard MT, Givskov M, Tolker-Nielsen T, Otzen DE, Nielsen PH 2013 Expression of Fap amyloids in Pseudomonas aeruginosa, P. fluorescens, and P. putida results in aggregation and increased biofilm formation. Microbiologyopen 2:365–382. doi:10.1002/mbo3.81. 23504942 PMC3684753

[27] Palmer KL, Mashburn LM, Singh PK, Whiteley M 2005 Cystic fibrosis sputum supports growth and cues key aspects of Pseudomonas aeruginosa physiology. J Bacteriol 187:5267–5277. doi:10.1128/JB.187.15.5267-5277.2005. 16030221 PMC1196007

[28] Dueholm MS, Otzen D, Nielsen PH 2013 Evolutionary insight into the functional amyloids of the pseudomonads. PLoS One 8:e76630. doi:10.1371/journal.pone.0076630. 24116129 PMC3792158

[29] Rouse SL, Hawthorne WJ, Berry JL, Chorev DS, Ionescu SA, Lambert S, Stylianou F, Ewert W, Mackie U, Morgan RML, Otzen D, Herbst FA, Nielsen PH, Dueholm M, Bayley H, Robinson CV, Hare S, Matthews S 2017 A new class of hybrid secretion system is employed in Pseudomonas amyloid biogenesis. Nat Commun 8:263. doi:10.1038/s41467-017-00361-6. 28811582 PMC5557850

[30] Dueholm MS, Nielsen SB, Hein KL, Nissen P, Chapman M, Christiansen G, Nielsen PH, Otzen DE 2011 Fibrillation of the major curli subunit CsgA under a wide range of conditions implies a robust design of aggregation. Biochemistry 50:8281–8290. doi:10.1021/bi200967c. 21877724 PMC3724407

[31] Kelly SM, Jess TJ, Price NC 2005 How to study proteins by circular dichroism. Biochim Biophys Acta 1751:119–139. doi:10.1016/j.bbapap.2005.06.005. 16027053

[32] Knowles TP, Waudby CA, Devlin GL, Cohen SI, Aguzzi A, Vendruscolo M, Terentjev EM, Welland ME, Dobson CM 2009 An analytical solution to the kinetics of breakable filament assembly. Science 326:1533–1537. doi:10.1126/science.1178250. 20007899

[33] Meisl G, Kirkegaard JB, Arosio P, Michaels TC, Vendruscolo M, Dobson CM, Linse S, Knowles TP 2016 Molecular mechanisms of protein aggregation from global fitting of kinetic models. Nat Protoc 11:252–272. doi:10.1038/nprot.2016.010. 26741409

[34] Cohen SI, Linse S, Luheshi LM, Hellstrand E, White DA, Rajah L, Otzen DE, Vendruscolo M, Dobson CM, Knowles TP 2013 Proliferation of amyloid-beta42 aggregates occurs through a secondary nucleation mechanism. Proc Natl Acad Sci U S A 110:9758–9763. doi:10.1073/pnas.1218402110. 23703910 PMC3683769

[35] Meisl G, Yang X, Hellstrand E, Frohm B, Kirkegaard JB, Cohen SI, Dobson CM, Linse S, Knowles TP 2014 Differences in nucleation behavior underlie the contrasting aggregation kinetics of the Abeta40 and Abeta42 peptides. Proc Natl Acad Sci U S A 111:9384–9389. doi:10.1073/pnas.1401564111. 24938782 PMC4084462

[36] Cohen SI, Arosio P, Presto J, Kurudenkandy FR, Biverstal H, Dolfe L, Dunning C, Yang X, Frohm B, Vendruscolo M, Johansson J, Dobson CM, Fisahn A, Knowles TP, Linse S 2015 A molecular chaperone breaks the catalytic cycle that generates toxic Abeta oligomers. Nat Struct Mol Biol 22:207–213. doi:10.1038/nsmb.2971. 25686087 PMC4595974

[37] LeVine H III. 2008 Thioflavine T interaction with synthetic Alzheimer's disease beta-amyloid peptides: detection of amyloid aggregation in solution. Protein Sci 2:404–410. doi:10.1002/pro.5560020312. PMC2142377 8453378

[38] Gaspar R, Meisl G, Buell AK, Young L, Kaminski CF, Knowles TP, Sparr E, Linse S 2017 Secondary nucleation of monomers on fibril surface dominates α-synuclein aggregation and provides autocatalytic amyloid amplification. Q Rev Biophys 50:e6. doi:10.1017/S0033583516000172. 29233218

[39] Chiti F, Dobson CM 2006 Protein misfolding, functional amyloid, and human disease. Annu Rev Biochem 75:333–366. doi:10.1146/annurev.biochem.75.101304.123901. 16756495

[40] Chiti F, Dobson CM 2017 Protein misfolding, amyloid formation, and human disease: a summary of progress over the last decade. Annu Rev Biochem 86:27–68. doi:10.1146/annurev-biochem-061516-045115. 28498720

[41] Buell AK, Galvagnion C, Gaspar R, Sparr E, Vendruscolo M, Knowles TP, Linse S, Dobson CM 2014 Solution conditions determine the relative importance of nucleation and growth processes in alpha-synuclein aggregation. Proc Natl Acad Sci U S A 111:7671–7676. doi:10.1073/pnas.1315346111. 24817693 PMC4040554

[42] Arosio P, Cukalevski R, Frohm B, Knowles TP, Linse S 2014 Quantification of the concentration of Abeta42 propagons during the lag phase by an amyloid chain reaction assay. J Am Chem Soc 136:219–225. doi:10.1021/ja408765u. 24313551

[43] Tartaglia GG, Pawar AP, Campioni S, Dobson CM, Chiti F, Vendruscolo M 2008 Prediction of aggregation-prone regions in structured proteins. J Mol Biol 380:425–436. doi:10.1016/j.jmb.2008.05.013. 18514226

[44] Tartaglia GG, Vendruscolo M 2008 The Zyggregator method for predicting protein aggregation propensities. Chem Soc Rev 37:1395–1401. doi:10.1039/b706784b. 18568165

[45] Poulsen LK, Ballard G, Stahl DA 1993 Use of rRNA fluorescence in situ hybridization for measuring the activity of single cells in young and established biofilms. Appl Environ Microbiol 59:1354–1360. 7685999 10.1128/aem.59.5.1354-1360.1993 PMC182089

[46] Yang L, Haagensen JA, Jelsbak L, Johansen HK, Sternberg C, Hoiby N, Molin S 2008 In situ growth rates and biofilm development of Pseudomonas aeruginosa populations in chronic lung infections. J Bacteriol 190:2767–2776. doi:10.1128/JB.01581-07. 18156255 PMC2293235

[47] Sleutel M, Van den Broeck I, Van Gerven N, Feuillie C, Jonckheere W, Valotteau C, Dufrêne YF, Remaut H 2017 Nucleation and growth of a bacterial functional amyloid at single-fiber resolution. Nat Chem Biol 13:902–908. doi:10.1038/nchembio.2413. 28628096 PMC5580806

[48] Liu X, Shen B, Du P, Wang N, Wang J, Li J, Sun A 2017 Transcriptomic analysis of the response of Pseudomonas fluorescens to epigallocatechin gallate by RNA-seq. PLoS One 12:e0177938. doi:10.1371/journal.pone.0177938. 28545064 PMC5435343

[49] Arita-Morioka K-I, Yamanaka K, Mizunoe Y, Tanaka Y, Ogura T, Sugimoto S 2018 Inhibitory effects of Myricetin derivatives on curli-dependent biofilm formation in Escherichia coli. Sci Rep 8:8452. doi:10.1038/s41598-018-26748-z. 29855532 PMC5981455

[50] Zhou Y, Smith DR, Hufnagel DA, Chapman MR 2013 Experimental manipulation of the microbial functional amyloid called curli. Methods Mol Biol 966:53–75. doi:10.1007/978-1-62703-245-2_4. 23299728 PMC3773212

