# Supplementary material for: Physical Determinants of Amyloid Assembly in Biofilm Formation
Source: mBio. 2019 Jan 8;10(1):e02279-18. doi: 10.1128/mBio.02279-18 (PMC6325246; doi:10.1128/mBio.02279-18)
Supplement: FIG S1 [file mBio.02279-18-sf001.pdf]

Figure S1

A

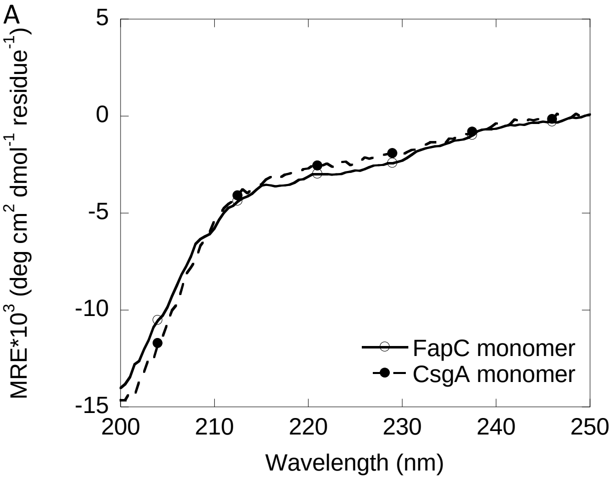

B

Structural component	FapC	CsgA
α-helix	4%	4%
β-sheet	48%	48%
Random coil	48%	48%

C D E F

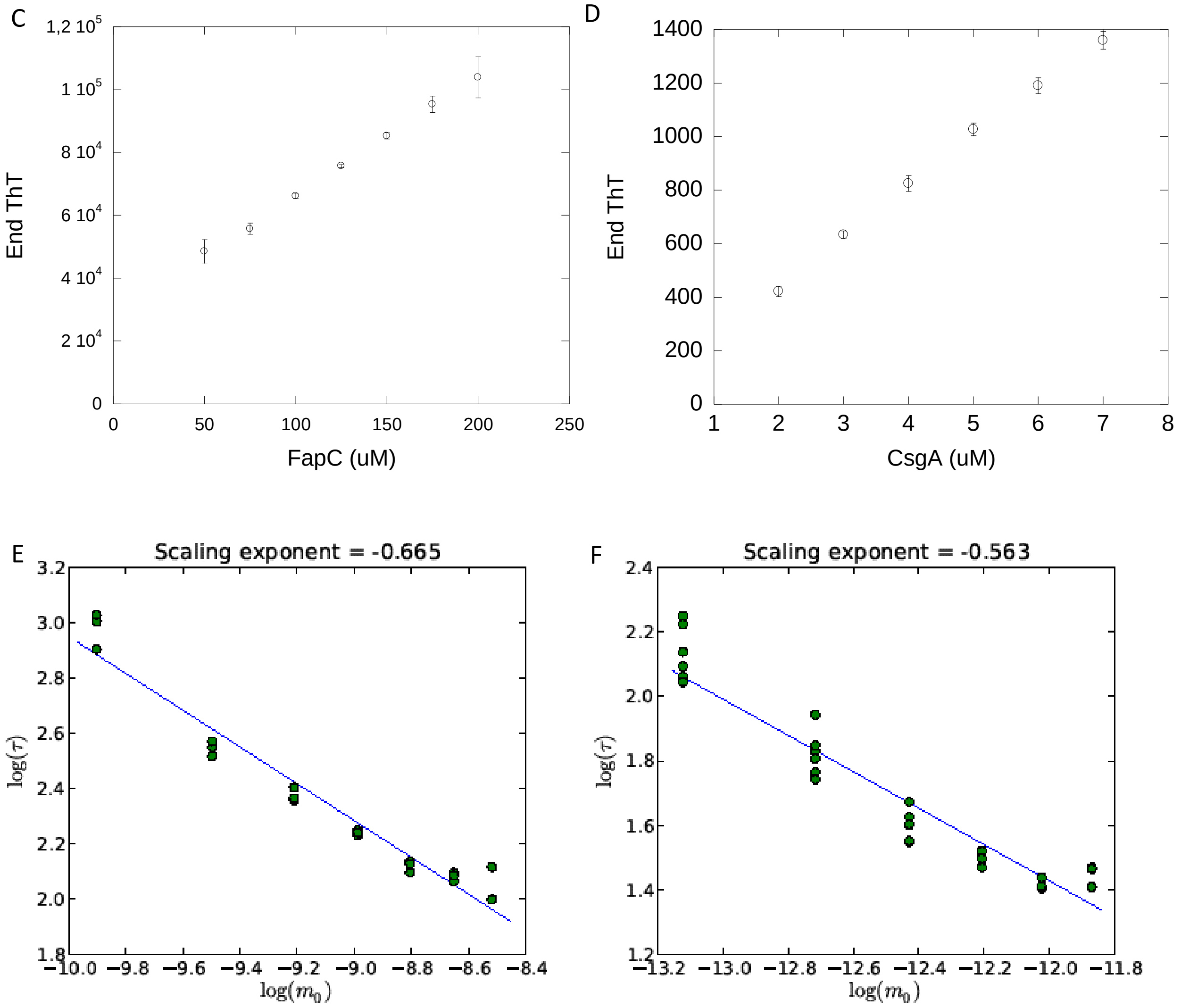

Figure S1: CD analysis of FapC and CsgA monomers and end-ThT scaling with initial monomer concentration and half-time analysis. Top panel: CD analysis of FapC and CsgA monomer structure. A: Far-UV CD spectra of FapC and CsgA monomers. B: Deconvolution of the Far-UV CD spectra shown in A using the K2d algorithm on the Dichroweb server (1, 2). The percentage contribution of various structural components is given for the monomeric form of both proteins. Middel panel: A linear scaling of the ThT fluorescence intensity with respect to the monomer protein concentration is seen for both proteins. The error-bars are shown in the plot. C: End-ThT level of FapC triplicates plotted against the initial monomer concentration. D: End-ThT level of six replicates of CsgA plotted against the initial monomer concentration. Bottom panel: The logarithm of the half time (the time it takes to reach half of the maximum ThT signal) of aggregation plotted against the logarithm of the initial monomer concentration. E: Half-time analysis of FapC kinetic data gives a straight line with the slope giving a scaling component of -0.665. F: Half-time analysis of CsgA kinetic data gives a straight line with the slope giving a scaling component of -0.563. The straight line in both plots indicates that the same dominant mechanism of fibril multiplication is the same for all monomer concentrations for both proteins.

The points in a log-log plot of the half-times (the time it takes to reach half the maximum ThT fluorescence) against initial monomer concentration fall on a straight line when analyzing kinetic data from both FapC and CsgA, confirming that the dominant mechanism for the aggregation process is likely to be the same in the range of monomer concentrations studied here.

1. Whitmore L, Wallace BA. 2008. Protein secondary structure analyses from circular dichroism spectroscopy: methods and reference databases. Biopolymers 89:392-400.
2. Whitmore L, Wallace BA. 2004. DICHROWEB, an online server for protein secondary structure analyses from circular dichroism spectroscopic data. Nucleic Acids Res 32:W668-73.
