# Supplementary material for: Physical Determinants of Amyloid Assembly in Biofilm Formation
Source: mBio. 2019 Jan 8;10(1):e02279-18. doi: 10.1128/mBio.02279-18 (PMC6325246; doi:10.1128/mBio.02279-18)
Supplement: TABLE S1 [file mBio.02279-18-st001.pdf]

Table S1

	FapC	CsgA
n_c [-]	1.31	1.12
$k_n k_+$ [$M^{-nc}h^{-2}$]	1.18×10^3	3.68×10^4
MRE	0.00115	0.00109

Table S1: Kinetic parameters for global fitting of the experimental data for FapC and CsgA to a nucleation-elongation model, where n_c is the reaction order of the primary nucleation process, k_n is the rate constant for the primary nucleation process and k_+ is the rate constant for the elongation of existing fibrils.
