# Supplementary material for: Physical Determinants of Amyloid Assembly in Biofilm Formation
Source: mBio. 2019 Jan 8;10(1):e02279-18. doi: 10.1128/mBio.02279-18 (PMC6325246; doi:10.1128/mBio.02279-18)
Supplement: TEXT S1 [file mBio.02279-18-s0001.pdf]

Text S1: Equations for global fitting to a nucleation-elongation model

The differential equations describing this system are:

$$\frac{dP}{dt} = k_n m(t)^{n_c} \tag{1}$$

$$\frac{dM}{dt} = 2\big(m(t)k_+ - k_{off}\big)P(t) \tag{2}$$

where k_n, k_+ and k_{off} are the rate constants of primary nucleation, elongation and depolymerisation, $m(t)$, $P(t)$ and $M(t)$ are the monomer, fibril number and fibril mass concentrations, respectively and n_c is the reaction order of primary nucleation. For a negligible depolymerisation rate, $k_{off} \ll k_+ m_0$, the closed form solution, based on Oosawa (3), is:

$$\frac{M}{m_{tot}} = 1 - \frac{m_0}{m_{tot}}\left(\frac{1}{\mu}\cosh\sqrt{\frac{n_c}{2}}\mu\lambda t + v\right)^{-\frac{2}{n_c}} \tag{3}$$

where the definitions of the parameters are

$$\lambda = \sqrt{2k_+ k_n {m_0}^{n_c}} \tag{4}$$

$$\alpha = \sqrt{\frac{k_+ n_c}{k_n {m_0}^{n_c}}}P_0 \tag{5}$$

$$\mu = \sqrt{1+\alpha^2} \tag{6}$$

$$v = \log(\alpha + \mu) \tag{7}$$

where m_{tot} is the total protein mass concentration and P_0 and m_0 are the initial fibril number and monomer concentrations at time t=0. In the unseeded case this depends only on the combined rate constant k_+k_n, not k_+ and k_n individually. The approximate scaling exponent is:

$$\gamma \approx -\frac{n_c}{2} \tag{8}$$

3. Oosawa F & Asakura S (1975) *Thermodynamics of the Polymerization of Protein* (Academic Press London).
