# Supplementary material for: Physical Determinants of Amyloid Assembly in Biofilm Formation
Source: mBio. 2019 Jan 8;10(1):e02279-18. doi: 10.1128/mBio.02279-18 (PMC6325246; doi:10.1128/mBio.02279-18)
Supplement: TEXT S2 [file mBio.02279-18-s0002.pdf]

Text S2: Integrated rate law for kinetics of aggregation at the cell surface

We derive here integrated rate laws for the irreversible polymerization reaction of protein molecules in solution on the surface of bacteria and show that in the limit of fast diffusion the resulting expressions yield the well-known early-time dependence of the aggregate mass predicted by conventional nucleated polymerization models of fibrillar growth. We consider the polymerization reaction between protein molecules in solution and reactive polymer ends attached to a surface. Denoting the elongation rate constant as k_+, the rate of change in reactant concentration as a function of time is described by the following equation

$$\frac{\partial m(\boldsymbol{x},t)}{\partial t} = -k_+ P(\boldsymbol{x},t) m(\boldsymbol{x},t), \qquad (1)$$

where $m(\boldsymbol{x},t)$ is the concentration of monomeric protein in solution at position $\boldsymbol{x}$ and time t, and $P(\boldsymbol{x},t)$ is the number concentration of aggregate ends. We consider here the situation in which the bacterial surface corresponds to the plane $x = 0$, so that polymer ends are distributed according to:

$$P(\boldsymbol{x},t) = P(t)\delta(x), \qquad (2)$$

where $P(t)$ now denotes the number of reactive polymer ends per unit area on the surface. The translational symmetry of the system parallel to the plane implies that time evolutions in the different dimensions are independent and an explicit time dependence enters only in the direction perpendicular to the surface, which here is denoted with x. By combing contributions from diffusion and reaction, the time evolution of the monomer concentration $m(x,t)$ is given by the following reaction-diffusion equation

$$\frac{\partial m(x,t)}{\partial t} = \left[D\frac{\partial^2}{\partial x^2} - k_+ P(t)\delta(x)\right] m(x,t). \qquad (3)$$

Constant number of aggregates on bacterial surface

We first consider the situation in which the number of aggregate ends distributed on the bacterial surface $x = 0$ is constant in time, such that:

$$k_+ P(t) = k_+ P(0) = k_{0.} \qquad (4)$$

Note that this situation corresponds to the case when an amount $P(0)$ of preformed seed aggregates is present at the beginning of the reaction and the protein molecules which add onto ordered amyloid fibrils attached to a surface adopt the configuration of the fibril template and can then promote the attachment of further proteins. A note on dimensionality: k_+ has units of $m^3 s^{-1}$ (or alternatively $M^{-1}s^{-1}$) and therefore k_0 has units of m/s, unusually for a rate constant. Reformulating the delta function in Eq. (3) as a boundary condition at $x = 0$, we need to solve

$$\frac{\partial m(x,t)}{\partial t} = D\frac{\partial^2 m(x,t)}{\partial x^2}, \qquad \left.\frac{\partial m(x,t)}{\partial x}\right|_{x=0} = k_0 m(x=0,t) \tag{5}$$

Introducing the Laplace transform

$$\hat{m}(x,s) = \int_0^\infty m(x,t)e^{-st}dt \tag{6}$$

allows rewriting the above equation for an initially homogeneous distribution $m(x,t=0) = m(0)$ as

$$s\hat{m}(x,s) - m(0) = D\frac{\partial^2 \hat{m}(x,s)}{\partial x^2}, \qquad \left.\frac{\partial \hat{m}(x,s)}{\partial x}\right|_{x=0} = k_0\hat{m}(x=0,s). \tag{7}$$

The solution is of the form

$$\hat{m}(x,s) = A(s)e^{-\sqrt{s}x/\sqrt{D}}\frac{m(0)}{s}, \tag{8}$$

where the constant of integration $A(s)$ is determined by the boundary condition at $x = 0$ as:

$$\left.\frac{\partial \hat{m}(x,s)}{\partial x}\right|_{x=0} = -\sqrt{s/D}\,A(s) = k_0\hat{m}(x=0,s) = k_0\left(A(s) + \frac{m(0)}{s}\right), \tag{9}$$

yielding the compact solution:

$$\hat{m}(x,s) = \frac{m(0)}{s}\left(1 - \frac{k_0 e^{-\sqrt{s}x/\sqrt{D}}}{\sqrt{s/D} + k_0}\right). \tag{10}$$

The time evolution of the concentration of reacting protein molecules in solution can be obtained by inverse Laplace transforming the above equation, yielding

$$m(x=0,t) = \frac{1}{2\pi i}\int_{c-i\infty}^{c+i\infty}\hat{m}(x=0,s)\,e^{st}ds = m(0)\,\mathrm{erfc}\left(\sqrt{\frac{k_0^2 t}{4D}}\right)e^{\frac{k_0^2 t}{4D}}, \tag{11}$$

where

$$\mathrm{erfc}(x) = \frac{2}{\sqrt{\pi}}\int_x^\infty e^{-\xi^2}d\xi \tag{12}$$

is the complementary error function. Finally, the time evolution of the mass concentration of aggregates on the surface of bacteria can be obtained upon integration of Eq. (11) as

$$M(t) = k_0\int_0^t m(x=0,\tau)d\tau = k_+ m(0)P(0)t - \frac{2k_+ m(0)P(0)}{3\sqrt{\pi D}}t^3 + \cdots \tag{13}$$

Note that in the limit of fast diffusion (or, equivalently, in the limit of early times), we recover the well-known early-time dependence of the aggregate mass predicted by conventional nucleated polymerization models of fibrillary growth

$$M(t) \xrightarrow{t\to 0} k_+ m(0) P(0) t. \tag{14}$$
