# Supplementary material for: Physical Determinants of Amyloid Assembly in Biofilm Formation
Source: mBio. 2019 Jan 8;10(1):e02279-18. doi: 10.1128/mBio.02279-18 (PMC6325246; doi:10.1128/mBio.02279-18)
Supplement: FIG S2 [file mBio.02279-18-sf002.pdf]

Figure S2

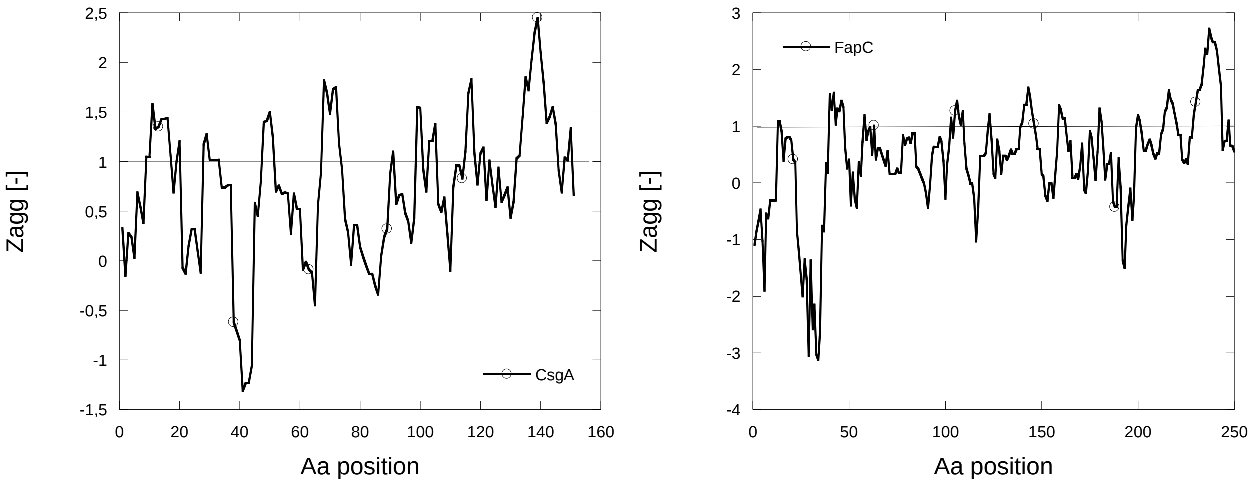

Figure S2:
For the kinetic analysis of CsgA concentrations an order of magnitude smaller than those used for FapC were used. This difference in the concentration ranges could be ascribed to the number of repeat units in each monomer. FapC has three repeat motifs which are incorporated into the β-sheet structure of the fibrils where CsgA has five such repeat regions. This increase number of repeat units in CsgA could result in an intrinsic elevated aggregation propensity as compared to FapC. This is also reflected in the overall aggregation propensity score assigned to each protein by the aggregation propensity prediction algorithm Zyggregator (4, 5). Using the software, the overall aggregation propensity, z_{agg}, of FapC is found to be 0.801±0.039 while for CsgA it is 0.928±0.046. A plot of the aggregation propensity along the amino acid sequence of FapC and CsgA can be seen in Supplementary Fig. 5. This could explain the need for higher concentrations of FapC as compared to CsgA in order for the aggregation to proceed

4. Tartaglia GG, *et al.* (2008) Prediction of aggregation-prone regions in structured proteins. *J Mol Biol* 380(2):425-436.
5. Tartaglia GG & Vendruscolo M (2008) The Zyggregator method for predicting protein aggregation propensities. *Chem Soc Rev* 37(7):1395-1401.
