# Supplementary material for: Physical Determinants of Amyloid Assembly in Biofilm Formation
Source: mBio. 2019 Jan 8;10(1):e02279-18. doi: 10.1128/mBio.02279-18 (PMC6325246; doi:10.1128/mBio.02279-18)
Supplement: FIG S3 [file mBio.02279-18-sf003.pdf]

Figure S3

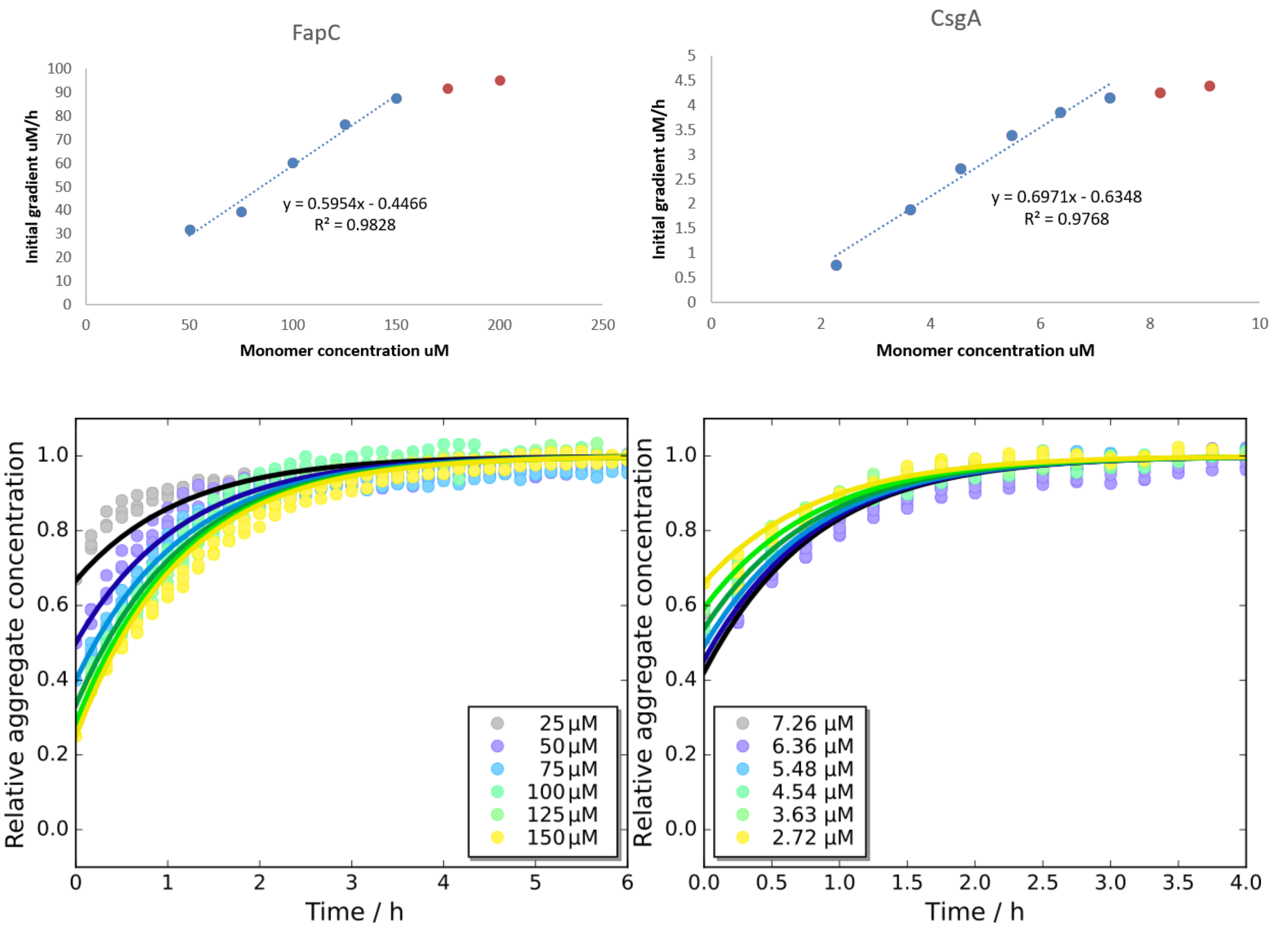

Figure S3: Fitting of seeded aggregation kinetic data of FapC. Top panel: Analysis of the initial gradient during seeded aggregation used to estimate the elongation rates. The initial gradient (first 30 min) of the above two datasets is plotted against the free monomer concentration. A straight line was fitted to these points, its slope proportional to the number of seed fibrils and the elongation rate constant. A slight effect of saturation of elongation can be seen at high concentrations, as observed frequently in such systems (6-8), hence these points (red) were excluded. Bottom panel: Global fitting of seeded aggregation kinetic data. The data was fitted to a nucleation elongation model with added seeds. The parameters k_+ and k_n were allowed to vary while their product k_+k_n was held constant at the value obtained for the unseeded data, see table SI2. For FapC a constant seed concentration of 50 µM and monomer concentrations ranging from 25 µM to 175 µM is used and for CsgA a constant seed concentration of 5.3 µM and monomer concentrations ranging from 2.72 µM to 9.08 µM is used.

6. Meisl G, *et al.* (2014) Differences in nucleation behavior underlie the contrasting aggregation kinetics of the Abeta40 and Abeta42 peptides. *Proc Natl Acad Sci U S A* 111(26):9384-9389.
7. Scheibel T, Bloom J, & Lindquist SL (2004) The elongation of yeast prion fibers involves separable steps of association and conversion. *Proc Natl Acad Sci U S A* 101(8):2287-2292.
8. Esler WP, *et al.* (2000) Alzheimer's disease amyloid propagation by a template-dependent dock-lock mechanism. *Biochemistry* 39(21):6288-6295.
