# Supplementary material for: Physical Determinants of Amyloid Assembly in Biofilm Formation
Source: mBio. 2019 Jan 8;10(1):e02279-18. doi: 10.1128/mBio.02279-18 (PMC6325246; doi:10.1128/mBio.02279-18)
Supplement: FIG S4 [file mBio.02279-18-sf004.pdf]

Figure S4

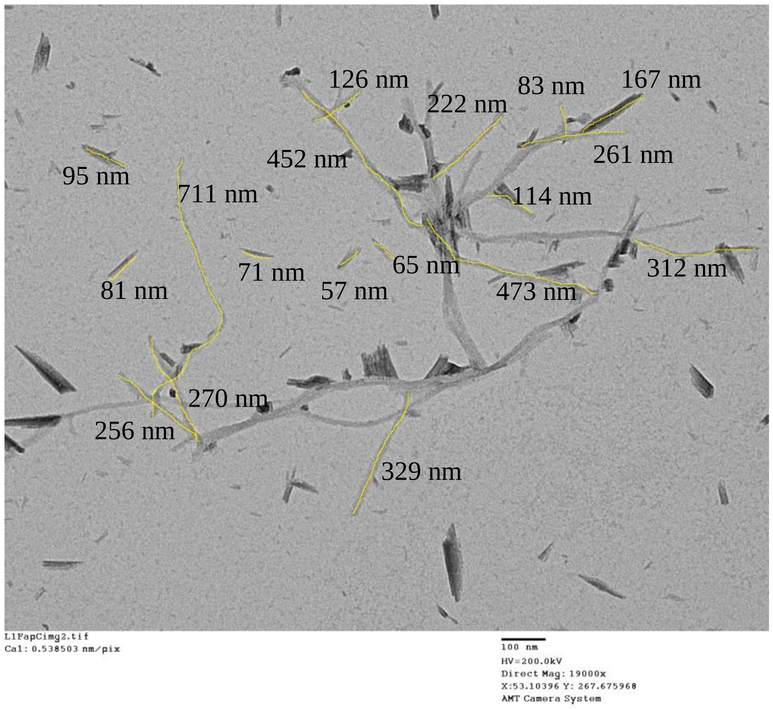

Figure S4: Measurements of fibril seed lengths from TEM image of sonicated fibril seeds. Scale bar indicates 100 nm. The image is of FapC fibril seeds and is representative of a larger set of fibril seeds analyzed.

Example of representative TEM image of fibril seeds length analysis with the length of individual seeds given in nm. The fibril dimensions extracted from the TEM images can be seen in Table S3. This images shows FapC fibril seeds.
