# Supplementary material for: Physical Determinants of Amyloid Assembly in Biofilm Formation
Source: mBio. 2019 Jan 8;10(1):e02279-18. doi: 10.1128/mBio.02279-18 (PMC6325246; doi:10.1128/mBio.02279-18)
Supplement: TABLE S2 [file mBio.02279-18-st002.pdf]

Table S2

	FapC	CsgA
M_0	50 μM	5.3 μM
P_0	39 nM	8 nM
n_c	1.31	1.21
k_n	1.07×10^{-4} M^{-nc+1} h^{-1}	5.06×10^{-4} M^{-nc+1} h^{-1}
k_+	1.11×10^{7} M h^{-1} (3100$M^{-1}s^{-1}$)	7.47×10^{7} M h^{-1} (21000 $M^{-1}s^{-1}$)
Mean residual error of fit	0.00067	0.00074

Table S2: Kinetic parameters for global fitting of the experimental data for seeded aggregation of FapC and CsgA to a nucleation-elongation model, where M_0 is the concentration of fibril seeds, P_0 is the number concentration of seed fibrils, n_c is the reaction order of the primary nucleation process, k_n is the rate constant for the primary nucleation process and k_+ is the rate constant for the elongation of existing fibrils. The values for the elongation rates are within a factor of 2 of those determined from the initial gradient analysis.
