# Supplementary material for: Physical Determinants of Amyloid Assembly in Biofilm Formation
Source: mBio. 2019 Jan 8;10(1):e02279-18. doi: 10.1128/mBio.02279-18 (PMC6325246; doi:10.1128/mBio.02279-18)
Supplement: TABLE S3 [file mBio.02279-18-st003.pdf]

Table S3

	FapC	CsgA
M_w	24998.4 Da	13092.6 Da
Length	238 nm ± 8 nm	156 nm ± 12 nm
Width	27 nm ± 2 nm	12.7 nm ± 0.6 nm
Height	8 nm ± 1 nm	5.0 nm ± 0.4 nm
#monomers pr. fibril seed	~1770	~630

Table S3: Dimensions of FapC and CsgA fibril seeds in nm extracted from TEM images. A total of 480 fibril seeds for both proteins have been analyzed. An average density of amino acids of 1.35 g/cm^3 is used in the calculations.
