# Supplementary material for: Physical Determinants of Amyloid Assembly in Biofilm Formation
Source: mBio. 2019 Jan 8;10(1):e02279-18. doi: 10.1128/mBio.02279-18 (PMC6325246; doi:10.1128/mBio.02279-18)
Supplement: TEXT S3 [file mBio.02279-18-s0003.pdf]

Text S3:

Estimation of length of growth time *in vivo*

Studies of biofilm formation *in vivo* have revealed that the EPS matrix is produced predominantly at the edge of the biofilm by cells in a region of constant thickness, which spread outwards as the central regions of the biofilm mature. Although studies have largely been performed on *B. subtilis* (9-12), in which the TasA protein forms amyloid fibrils within the biofilm, similar results have been obtained for *E. coli* (13).

The quantity of interest in the context of this work is the time a small region of the biofilm takes to mature. This is estimated by dividing the thickness of the growing region by the speed of expansion. For *B. subtilis*, whose biofilms are well studied in terms of their biophysical properties, two works performed under different conditions report (1) a biofilm spreading speed of 250 µm/h with a growing region thickness of 2 mm (14) (where the growing region was identified by monitoring gene expression) and (2) an average speed of approximately 10 µm/h with a thickness of approximately 100 µm (15) (where the growing region was identified by image differencing of phase contrast images of the biofilm taken in 10 min intervals). In both cases the time spent in the growing region is therefore approximately 10 h. For *E. coli* a study on the local mechanics within a biofilm reveals a region of high stiffness in the center of the biofilm, surrounded by a region of varying stiffness, which we assume corresponds to the growing region (16). The growing region has a thickness of 25 µm and moves by 20 µm in 24 h. Hence the time spent in the growing region is 30 h. Given surprising the similarity of the values of the time spent in the growing region in these very different systems, we believe 10 h is a good general order of magnitude estimate.

To compare this timescale of biofilm spread to that of amyloid formation measured here *in vitro*, we thus assume that during this 10 h period a continuous fibril network must assemble. In order to produce a continuous biofilm, the fibrils produced by one cell have to bridge the distance to the neighboring cell. Such a distance will be comparable to the size of the cell itself, which is approximately 1 µm. Hence, given a 10 h period to bridge this gap, a growth rate on the order of 100 nm/h or 0.03 nm/s is required. From measurements of the dimensions of the fibrils by TEM (see Fig. S4 and Table S3), we estimate that there are approximately 10 monomers per nm of fibril length, giving 0.3 monomers/s as the growth rate required to bridge the intracellular gap within 10 h. Alternative models and their shortcomings are discussed below.

Estimating the extracellular *in vivo* concentration of amyloid forming protein

We have purified 10 mg native FapC fibrils from 50 g bacterial cell pellet. Assuming that 50 g cell pellet corresponds to 50 mL biofilm, this yields a FapC concentration of 0.2 mg/mL or approximately 10 µM FapC monomer equivalents. Previously 1 mg amyloid CsgA was produced pr. g cell pellet. Using the same calculations as with FapC, this gives 1 mg/mL or approximately 40 µM CsgA. The cell pellet is probably somewhat denser than the biofilm; however, as we consider concentrations in the extracellular volume the bacterial cell volume does not contribute. This constitutes the total concentration of amyloid forming protein. The free monomer concentration at any point during assembly is likely much lower than this. Therefore 10 µM will be a conservative upper bound on the average monomer concentration during growth. To provide an additional consistency check of the value obtained for the total amount of protein, we use the fibril dimensions measured *in vitro* to predict fibril density on the biofilm. A fibril-length of 1 µm corresponds to approximately 10000 monomers, hence for a fibril mass concentration on the order of 10 µM, the fibril number concentration is 1 nM, i.e. 10^{-6} moles per m^3. This corresponds to 6×10^{17} fibrils per m^3, or approximately one fibril per μm^3. Given that a significant part of the volume will be taken up by cells and that 10 µM is a lower bound on the total protein concentration due to losses during purification, this estimate is consistent with having a few fibrils per cell in the biofilm.

Alternative models for *in vivo* growth

Based on several biophysical studies of biofilm formation we assumed that cell spread precedes the spread of amyloid fibrils in the above description. However, it is interesting to consider alternative models in which amyloid spreads before cells do and determine whether the rate constants observed *in vitro* are still consistent with such models. In the simplest model, the fibrils spread by direct elongation from the edge of the biofilm; the speed of spread of amyloid is then simply given by the elongation rate. Biofilms spread at a rate of between 1 µm/h (16) and >100 µm/h (14, 15), which, given an elongation rate constant of 10^4 $M^{-1}s^{-1}$, would require free monomer concentrations between 100 µM and 10 mM under our *in vitro* conditions. As shown by Sleutel et al. (17), the presence of surfaces can significantly increase the speeds of growth, but their fastest observed speeds of 5nm/s, i.e. 18 µm/h are still below those of some of the faster growing biofilms observed *in vivo*.

Alternatively, spread of aggregates might occur via multiplication and diffusion of newly formed fibrils, as was observed for example for the aggregation of Aβ42 in microdroplets (18). In this case

the spreading velocity is given by $2\sqrt{D\kappa}$ where D is the diffusion coefficient and κ depends on the rate constants of elongation and multiplication. As we do not observe any multiplication processes *in vitro*, we can only obtain an upper bound on the rate of multiplication. This is done by assuming multiplication occurs via fragmentation and that the peak multiplication rate is less than the peak primary nucleation rate. The primary nucleation rate peaks at the beginning of the experiment, where it is given by $k_n m_0^{nc}$. The fragmentation rate peaks at the end of the experiment and is given by $k_- m_0$ (assuming all monomer is converted to its aggregated form at the end of the experiment). κ is given by $\sqrt{2k_+k_-m_0'}$ where m_0' is the monomer concentration present in the biofilm and hence the velocity of spread

$$v = 2\sqrt{D}\sqrt[4]{2k_+k_-}\sqrt[4]{m_0'}. \tag{15}$$

By applying the above condition on the peak rates, we obtain

$$k_+k_- = k_+k_n m_0^{(n_n-1)}, \tag{16}$$

where we use the maximum monomer concentration used *in vitro* for m_0 to obtain an upper bound. Combining with the above and rearranging yields

$$m_0' = \frac{v^4}{32D^2k_+k_n m_0^{(n_n-1)}}. \tag{17}$$

Assuming a diffusion coefficient of 10^{-12} m^2s^{-1} (corresponding to a hydrodynamic radius of approximately 500 nm) we obtain that picomolar concentrations of monomer are sufficient to achieve biofilm spreading rates of 1 µm/h whereas milimolar concentrations are required for spreading rates of 100 µm/h. The large range of concentration values results from the fact that the spreading rate only weakly depends on the monomer rate (with the fourth root of m_0).

In conclusion, the former model (direct elongation) requires significantly higher free monomer concentrations or other mechanisms speeding up growth to achieve the same rate as the model in the main text. For the latter model (self-replication and diffusion), the required monomer concentrations span a large range of values, and hence this model cannot be excluded based on this analysis with our estimation of an upper bound for the fragmentation rate. However, given that the organisms produce proteins that fulfill the specific task of nucleating fibril growth and given the fact that cells appear to produce their fibrils relatively independently of their neighbors, a self-replication and diffusion model seems less likely to be relevant *in vivo*.

9. Liu J, Prindle A, Humphries J, Gabalda-Sagarra M, Asally M, Lee DY, Ly S, Garcia-Ojalvo J, Suel GM. 2015. Metabolic co-dependence gives rise to collective oscillations within biofilms. Nature 523:550-4.
10. Srinivasan S, Vladescu ID, Koehler SA, Wang X, Mani M, Rubinstein SM. 2017. Matrix Production In Bacillus subtilis Biofilms Is Localized To A Propagating Front. bioRxiv:129825.
11. Angelini TE, Roper M, Kolter R, Weitz DA, Brenner MP. 2009. Bacillus subtilis spreads by surfing on waves of surfactant. Proc Natl Acad Sci U S A 106:18109-13.
12. Seminara A, Angelini TE, Wilking JN, Vlamakis H, Ebrahim S, Kolter R, Weitz DA, Brenner MP. 2012. Osmotic spreading of Bacillus subtilis biofilms driven by an extracellular matrix. Proc Natl Acad Sci U S A 109:1116-21.
13. Galy O, Latour-Lambert P, Zrelli K, Ghigo JM, Beloin C, Henry N. 2012. Mapping of bacterial biofilm local mechanics by magnetic microparticle actuation. Biophys J 103:1400-8.
14. Srinivasan S, *et al.* (2017) Matrix Production In Bacillus subtilis Biofilms Is Localized To A Propagating Front. *bioRxiv*:129825.
15. Liu J, *et al.* (2015) Metabolic co-dependence gives rise to collective oscillations within biofilms. *Nature* 523(7562):550-554.
16. Galy O, *et al.* (2012) Mapping of bacterial biofilm local mechanics by magnetic microparticle actuation. *Biophys J* 103(6):1400-1408.
17. Sleutel M, Van den Broeck I, Van Gerven N, Feuillie C, Jonckheere W, Valotteau C, Dufrene YF, Remaut H. 2017. Nucleation and growth of a bacterial functional amyloid at single-fiber resolution. Nat Chem Biol 13:902-908.
18. Knowles TP, *et al.* (2011) Observation of spatial propagation of amyloid assembly from single nuclei. *Proc Natl Acad Sci U S A* 108(36):14746-14751.
